# Occupational exposure to diesel exhausts and liver and pancreatic cancers: a systematic review and meta-analysis

**DOI:** 10.1007/s10654-024-01099-4

**Published:** 2024-01-30

**Authors:** Michele Sassano, Giulia Collatuzzo, Federica Teglia, Paolo Boffetta

**Affiliations:** 1 Department of Medical and Surgical Sciences, University of Bologna, Bologna, Italy; 2 Stony Brook Cancer Center, Stony Brook University, Stony Brook, NY USA; 3 Department of Family, Population and Preventive Medicine, Renaissance School of Medicine, Stony Brook University, Stony Brook, NY USA

**Keywords:** Occupation, Diesel, Liver cancer, Pancreatic cancer, Meta-analysis

## Abstract

**Background:**

Diesel exhaust (DE) is human carcinogen with sufficient evidence only for lung cancer. Systematic evidence on other cancer types is scarce, thus we aimed to systematically review current literature on the association between occupational DE exposure and risk of liver and pancreatic cancers.

**Methods:**

We performed a systematic literature review to identify cohort studies on occupational DE exposure and risk of cancers other than lung. We computed pooled relative risks (RRs) and corresponding 95% confidence intervals (CIs) for liver and pancreatic cancers using DerSimonian and Laird random-effects model.

**Results:**

Fifteen studies reporting results on pancreatic cancer and fourteen on liver cancer were included. We found a weakly increased risk of pancreatic cancer in workers exposed to DE (RR: 1.07, 95% CI: 1.00, 1.14), mainly driven by results on incidence (RR: 1.11, 95% CI: 1.02, 1.22). As for liver cancer, results were suggestive of a positive association (RR: 1.09; 95% CI: 0.99, 1.19), although a significant estimate was present in studies published before 2000 (RR: 1.41; 95% CI: 1.09, 1.82). We found no compelling evidence of publication bias.

**Conclusions:**

Our findings suggest an association between occupational DE exposure and liver and pancreatic cancer. Further studies with detailed exposure assessment, environmental monitoring data, and appropriate control for confounders are warranted.

**Supplementary Information:**

The online version contains supplementary material available at 10.1007/s10654-024-01099-4.

## Introduction

With an estimated 905,677 and 495,773 new cases worldwide, liver and pancreatic cancers currently represent the sixth and twelfth most common cancer types, respectively [1–3]. In addition, liver and pancreatic cancers were the third (*n* = 830,180) and the seventh (*n* = 466,003) leading causes of cancer death globally, respectively, in 2020 [1–3]. Although the highest incidence rates of liver cancer are observed in Asian countries, they declined between 1978 and 2012 in this region, while they increased over the same time period in many countries of Europe, the Americas, and Oceania [4]. In addition, the number of new cases of liver cancer is predicted to increase by 55% by the year 2040 [5]. Similarly, an increase of incidence rates of pancreatic cancer between 1990 and 2017 worldwide has been reported, with the largest burden recorded in high-income countries [6]. 

Among various potentially relevant environmental and occupational risk factors [7–9], previous research also focused on the relationship between exposure to diesel exhausts (DE) and cancer. Indeed, DE emissions contain a relevant number of suspected or confirmed carcinogens that could affect human health, including polycyclic aromatic hydrocarbons, nitroarenes, and 3-nitrobenzathrone [10–12]. DE emission has been classified as a Group 1 carcinogen by the International Agency for Research on Cancer (IARC), with sufficient evidence for lung cancer [11–13]. 

Previous meta-analyses of epidemiological studies mainly focused on the effect of DE exposure on lung cancer risk, with older ones showing a positive association between the two [14, 15] and more recent ones reporting more conflicting results [16, 17]. In addition, evidence of an increased risk of bladder cancer among individuals exposed to DE has been reported [18]. To date, two meta-analyses evaluating the association between DE exposure and pancreatic cancer have been published. The first one, not focused solely on DE but extended to the evaluation of the effect of 23 different chemicals in the workplace, reported a lack of association; [19] this conclusion was confirmed by a subsequent meta-analysis [20]. However, these meta-analyses included both cohort and case-control studies, and the latter have limitations mainly related to selection of participants and occupational exposure assessment [21]. In addition, both meta-analyses were not published recently, thus an update would be beneficial for the understanding of the potential carcinogenicity of DE on these organs. To our knowledge no previous meta-analysis evaluated the risk of liver cancer among workers in relation to their exposure to DE.

Thus, we aimed to summarize the current evidence deriving from cohort studies and related to the potential association of occupational DE exposure with liver and pancreatic cancers.

## Methods

We carried out a systematic review and reported it herein in accordance with the recommendations of the Preferred Reporting Items for Systematic Reviews and Meta-Analyses (PRISMA) statement [22]. Its protocol was registered in the PROSPERO database (registration number CRD42022352729).

We included all primary cohort or nested case-control studies on the association between occupational DE exposure and cancer types other than lung cancer from the last IARC Monograph on this topic [12]. Furthermore, we searched reference lists of studies in the IARC Monograph, and also performed a systematic search on Pubmed in order to identify other relevant studies investigating this association published after 2012, the last year included in the review in the abovementioned Monograph [12]. The search strategy was developed according to the Patients, Exposure, Comparator, Outcomes, Study design (PECOS) framework [23], with the following structure:

Population (P): workers in multiple industrial settings,

Exposure (E): occupational DE exposure,

Comparator (C): individuals not exposed to diesel,

Outcomes (O): incidence or mortality of cancer types other than lung cancer,

Study design (S): industry-based cohort.

Hence, the following search string was adopted for the search: *(diesel OR miner OR garage OR railway OR ((truck OR bus) AND driver) OR (heavy equipment OR docker)) AND (cancer OR neoplasm)*. The search was concluded in June 2021.

Two researchers (G.C., F.T.) independently screened titles and abstracts of identified articles. Thus, full texts of retained articles were retrieved, read, and included if relevant, following the same procedure. In addition, a manual search of reference lists of included articles and previous systematic reviews to identify additional studies was carried out. Any disagreements were solved by discussion.

The present systematic review is part of a larger project including all cancer types other than lung. Thus, during the phases of the study selection process, we included identified articles if they were: (1) English-written peer-reviewed reports with original data based on workers exposed to DE, (2) cohort studies or case-control studies nested within a cohort, (3) studies investigating the association between occupational DE exposure and incidence and/or mortality of primary cancer of sites other than lungs (4) studies reporting or allowing the computation from available data of a relative measure of association, including relative risk (RR), hazard ratio (HR), standardized mortality ratio (SMR), and standardized incidence ratio (SIR).

The following studies were excluded: case-control studies not nested in a cohort, cross-sectional studies, and descriptive studies, other systematic reviews or meta-analyses, conference proceedings, theses, letters to the editor, commentaries, book chapters, studies assessing only non-occupational exposures, and studies with no reference or mention to DE exposure. Whenever study populations overlapped across different reports, we included the most informative study, typically the one with the largest number of cases for the outcome of interest. Studies with less than 10% overlap of study populations were considered independent.

Two researchers (G.C., F.T.) independently extracted the following information from included studies: author details, publication year, country, study period, type of cohort (retrospective, prospective), type of reference (internal, external), type of workers, person-years of observation time, sample size, participants’ sex, outcome (incidence, mortality), type of cancer and International Classification of Diseases (ICD) code, number of cases, and main results, including adjustment factors.

Thus, we eventually included in the present meta-analysis only studies reporting data on liver and biliary tract or pancreatic cancers, which represent the focus of this report.

Quality assessment of included studies was performed independently by two researchers (G.C., F.T.) according to a modified version of the Critical Appraisal Skills Programme (CASP) checklist for cohort studies [24]. The modified scale is divided into 3 sections, including ‘are the results of the study valid?’ (6 items), ‘what are the results?’ (2 items), and ‘will the results help locally?’ (3 items). Each item received the maximum score if the researchers considered the quality of the content high, with a total score ranging between 0 and 14. For each study, the final total score was the average of those assigned by the two reviewers. Further details regarding the checklist are reported in Supplementary Table 1.

All relative measures of association described above were considered approximations of RRs. Thus, study-specific estimates of RRs and corresponding 95% confidence intervals (CIs) were pooled using DerSimonian and Laird random-effects model [25]. In order to assess statistical heterogeneity between studies we used the I^2^ statistic [26, 27]. We included in the analyses the most adjusted estimates provided by original studies. First we carried out the analysis combining data on both incidence and mortality (including estimates on incidence for studies reporting both of them), and then separate analyses for each outcome. The rationale for including both incidence and mortality as the outcome in the same meta-analysis derived from the consideration of mortality as a valid indicator of incidence, since the case-fatality rate for both cancer types considered is high [28]. According to the latest results from the Surveillance, Epidemiology, and End Results (SEER) program, 5-year survival rates have been estimated to be lower than 22% for liver cancer and 12% for pancreatic cancer [29]. Separate results from a single study (e.g., for specific strata or separate estimates for liver and gallbladder cancers) were combined using an inverse variance fixed-effects model, if needed, and then pooled with results from other studies as described above. Since a number of studies did not report separate results for liver and other biliary tract organs (including gallbladder), these cancer types were only considered together in the meta-analysis.

We performed a sensitivity analysis by excluding one study at a time in order to assess the individual influence of study-specific estimates on the results. Furthermore, we carried out subgroup analyses according to study participants’ sex, study region (North America, Europe), study quality (≤ median value among studies with data on the same cancer type, > median value), and publication year (before 2000, 2000 or later). Estimates from studies with a proportion of same-sex participants of 90% or higher were considered sex-specific.

Lastly, we assessed the occurrence of publication bias by using contour-enhanced funnel plot and Egger’s test [30–32]. 

All analyses were performed using STATA software version 17.0 (StataCorp LLC, College Station, Texas, USA).

## Results

### Characteristics of included studies

**Fig. 1:**
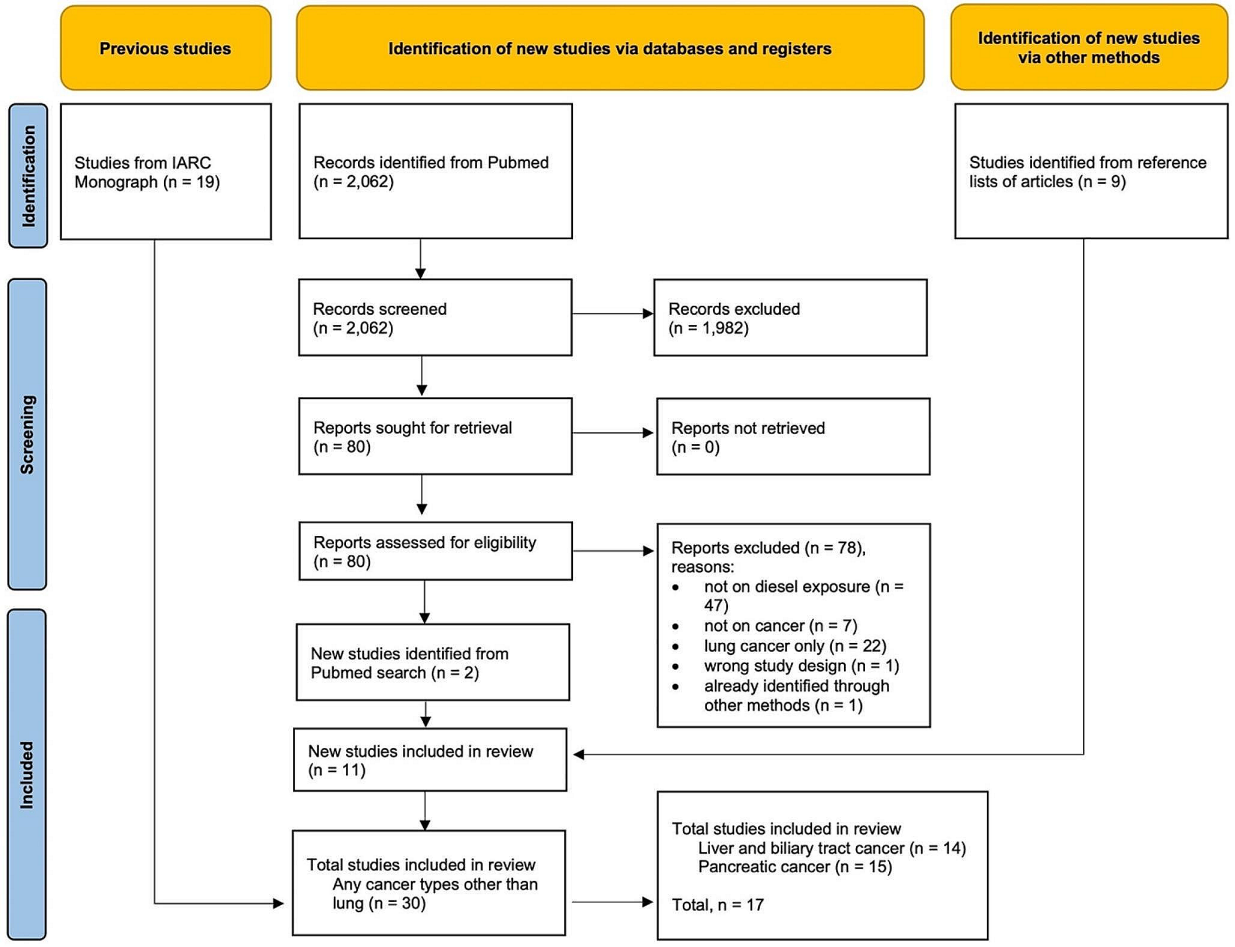
Flow diagram of the study selection process

The study selection process is represented in Fig. 1. A total of 19 non-overlapping studies were selected from the IARC Monograph [12]. The search of studies reported after 2012 included a total of 2,062 records, 1,982 of them were excluded in the screening phase. Overall, 80 full-text articles were assessed and 78 of them were excluded, eventually leading to the inclusion of 2 studies. In addition, 9 studies were identified from the lists of references of the studies in the IARC Monograph, leading to a total of 30 studies. Of these, 17 provided results for liver or pancreatic cancer [33–49]. 

Main characteristics of included studies are reported in Table 1. In particular, they were published between 1983 and 2020, with 9 (52.9%) of them conducted in Europe [35, 37, 40, 42, 43, 46–49] and 8 (47.1%) in Northern America [33, 34, 36, 38, 39, 41, 44, 45]. All included studies had a cohort design, while no nested case-control studies were retrieved.

**Table 1:** Main characteristics of included studies

Authors, year [ref]	Country	Study period	Type of cohort	Type of reference	Type of workers	Person-years	Sample size	Sex, male (%)	Outcome	Type of cancer (ICD code)	Number of cases	Results, estimate (95% confidence interval)	Variables controlled for	CASP score
Howe GR et al., 1983 [33]	Canada	1965–1977	Retrospective	external	Railway workers	290,186	43,826	100	M	Liver (ICD-7: 155, ICD-8: 155–156)	68	SMR: 0.99	Age (standardization)	9
										Pancreas (ICD-7: 157, ICD-8: 157)	197	SMR: 0.93		
Rushton L et al., 1983 [47]	United Kingdom	1968–1975	Retrospective	external	Bus garage workers	50,008		100	M	Liver and gallbladder	6	SMR: 1.47	Age (standardization)	7
										Pancreas	9	SMR: 0.92		
Wong O et al., 1985 [44]	USA	1964–1978	Retrospective	external	Heavy construction equipment operators	372 525.6	34,156	100	M	Liver (ICD-7: 155–156)	23	SMR: 1.67 (1.06, 2.50)	Age, race/ethnicity, and sex (standardization)	8
										Pancreas (ICD-7: 157)	47	SMR: 0.97 (0.71, 1.29)		
Boffetta P et al., 1988 [34]	USA	1982–1984	Prospective	internal	Mixed	939,817	476,648	100	M	Liver and biliary tract (ICD-9: 155–156)	Exposed: 7	RR: 1.13	Age, smoking, other occupational exposures (standardization)	10
									M	Pancreas (ICD-9: 157)	Total: 227, exposed: 27	RR: 1.39		
Bender AP et al., 1989 [41]	USA	1945–1984	Retrospective	external	highway maintenance workers		4849	100	M	Pancreas (ICD-9: 157)	17	SMR: 0.89 (0.52, 1.42)	(standardization)	10
Gustavsson P et al., 1990 [48]	Sweden	1952–1986	Retrospective	external	Bus garage workers		695	100	M	Liver (ICD-8: 155)	3	SMR: 1.50 (0.31, 4.93)	Age, sex (standardization)	10
										Pancreas (ICD-8: 157)	2	SMR: 0.46 (0.05, 1.65)		
		1958–1984							I	Liver (ICD-8: 155)	2	SIR: 0.88 (0.10, 3.18)		
										Pancreas (ICD-8: 157)	2	SIR: 0.57 (0.06, 2.07)		
Rafnsson V and Gunnarsdòttir H, 1991 [40]	Iceland	1951–1988	Retrospective	external	truck drivers	28,788	868	100	M	Pancreas (ICD-7: 157)	8	SMR: 1.50 (0.65, 2.96)	Age (standardization)	9
Guberan E et al., 1992 [42]	Switzerland	1949–1986	Retrospective	external	Professional drivers		1726	100	M	Liver (ICD-8: 155)	6	SMR: 0.88 (0.38, 1.74)	Age (standardization)	10
										Gallbladder (ICD-8: 156)	2	SMR: 1.18 (2.1, 3.7)		
										Pancreas (ICD-8: 157)	7	SMR: 0.91 (0.43, 1.71)		
		1970–1986							I	Liver (ICD-8: 155)	3	SMR: 0.48 (0.13, 1.25)		
										Gallbladder (ICD-8: 156)	4	SMR: 2.86 (0.98, 6.54)		
										Pancreas (ICD-8: 157)	3	SMR: 0.59 (0.16, 1.52)		
Van Den Eeden SK and Friedman GD, 1993 [45]	USA	1964–1988	Retrospective	internal	Mixed		160,230	46	I	Liver	108	HR: 1.11 (0.44, 2.77)	Age, gender, education, race/ethnicity, smoking status, duration, and amount	12
										Gallbladder	80	HR: 0.64 (0.20, 2.08)		
										Pancreas	426	HR: 1.42 (0.86, 2.34)		
Soll-Johanning H et al., 1998 [35]	Denmark	1943–1992	Retrospective	external	bus drivers and tramway employees	386,395	18,120	100	I	Liver	40	SIR: 1.60 (1.20, 2.20)	Age, sex (standardization)	10
										Pancreas	57	SIR: 1.20 (0.90, 1.60)		
								0			2	SIR: 1.60 (0.20, 5.90)		
Boffetta P et al., 2001 [46]	Sweden	1971–1989	Retrospective	external	Mixed	5,305,895		100	I	Liver (ICD-7: 155)	1196	SIR: 1.03 (0.97, 1.09)	Age (standardization)	11
										Pancreas (ICD-7: 157)	1859	SIR: 1.05 (1.00, 1.10)		
						240,586		0		Liver (ICD-7: 155)	42	SIR: 0.95 (0.69, 1.29)		
										Pancreas (ICD-7: 157)	47	SIR: 1.09 (0.80, 1.45)		
Järvholm B and Silverman D, 2003 [43]	Sweden	1971–1995	Retrospective	external	Heavy construction equipment operators		14,364	100	I	Liver (ICD-7: 155)	15	SIR: 0.94	Age (standardization)	11
					Drivers		6364				11	SIR: 0.98		
Pukkala E et al., 2009 [37]	Denmark, Finland, Iceland, Norway, Sweden	1960–2005	Retrospective	external	Engine operators		Around 14.9 million	100	I	Liver	403	SIR: 1.13 (1.02, 1.25)	Age (standardization)	13
								0			10	SIR: 1.39 (0.67, 2.56)		
								100		Gallbladder	175	SIR: 1.06 (0.91, 1.23)		
								0			8	SIR: 0.67 (0.29, 1.31)		
								100		Pancreas	970	SIR: 1.05 (0.99, 1.12)		
								0			27	SIR: 0.98 (0.64, 1.42)		
Birdsey J et al., 2010 [36]	USA	1989–2004	Retrospective	external	truck drivers		156,241	94	M	Liver and biliary tract	43	SMR: 0.72 (0.52, 0.97)	Age, race/ethnicity, and sex (standardization)	9
Merlo DF et al., 2010 [49]	Italy	1970–2005	Retrospective	external	bus drivers, bus maintenance workers, and white-collar workers	230,009	9184	100	M	Liver (ICD-9: 155)	59	SMR: 0.84 (0.65, 1.09)	Age (standardization)	11
										Pancreas (ICD-9: 157)	41	SMR: 1.00 (0.74, 1.36)		
Attfield MD et al., 2012 [39]	USA	1960–1997	Prospective	external	Non-metal miners	264,661	12,270	96	M	Pancreas (ICD-9: 157)	30	SMR: 1.12 (0.76, 1.60)	state, race/ethnicity, and sex (standardization)	10
										Liver and biliary tract (ICD-9: 155–156)	16	SMR: 1.17 (0.67, 1.89)		
Singh S et al., 2020 [38]	Canada	1991–2011	Retrospective	internal	Mixed (Other installers, repairers and servicers and material handlers + transport and heavy equipment operation and related maintenance occupations)		122,615	NR, both sexes	I	Pancreas	Exposed: 210	HR: 1.47 (1.18, 1.83)	Age, sex, education level, income, immigration status, province of residence	9

NR: not reported, I: incidence, M: mortality, HR: hazard ratio, SMR: standardized mortality ratio, SIR: standardized incidence ratio, RR: relative risk, ICD: International Classification of Diseases

Fourteen studies reported data on liver cancer [33–37, 39, 42–49] and 15 on pancreatic cancer [33–35, 37–42, 44–49]. . Among those on liver cancer, 5 (35.71%) studies focused on incidence only, 7 (50.00%) on mortality, and 2 (14.29%) examined both. Similarly, as for studies on pancreatic cancer, most (*n* = 8, 53.33%) evaluated only mortality, 5 (33.33%) incidence, and 2 (13.33%) both.

The median CASP score was equal to 10 (interquartile range, IQR: 9, 11) both when considering studies reporting results on liver cancer and those on pancreatic cancer.

### Meta-analysis

#### Liver cancer

**Fig. 2:**
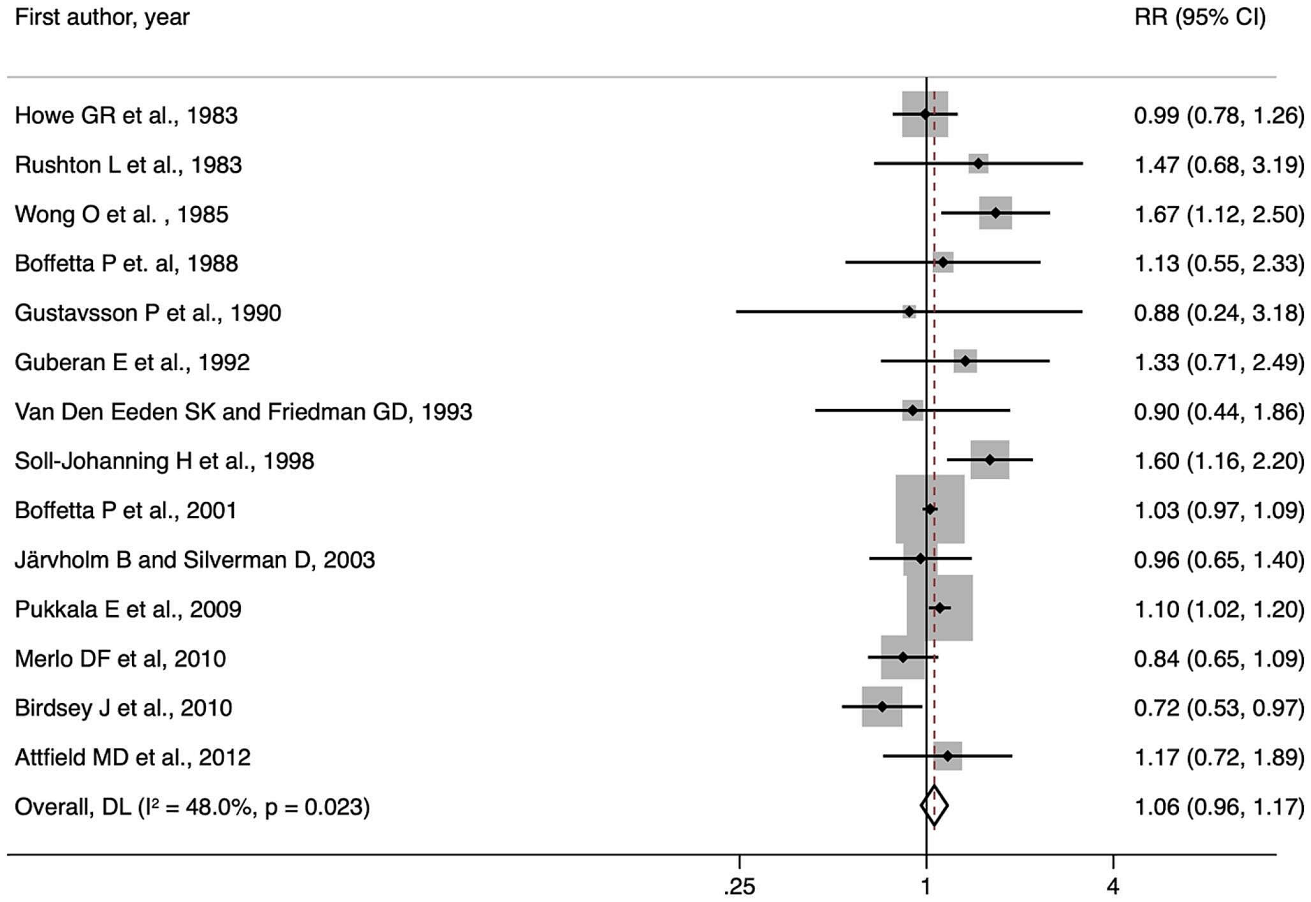
Results of the meta-analysis on the association between occupational exposure to diesel exhausts and liver and biliary tract cancer incidence and mortality combined

The results of the meta-analysis on incidence and mortality combined are reported in Fig. 2, showing a non-significant association between occupational DE exposure and liver cancer (RR: 1.06; 95% CI: 0.96, 1.17).

Results remained similar when omitting one study at a time (Supplementary Fig. 1). In subgroup analyses (Table 2), results remained similar among male and female study participants. Stronger but not -significant associations were observed when considering studies carried out in Europe (RR: 1.07; 95% CI: 0.97, 1.19) or with a CASP score higher than the median (RR: 1.04; 95% CI: 0.98, 1.11), while an increased risk of liver cancer associated with occupational DE exposure was found among studies published before the year 2000 (RR: 1.27; 95% CI: 1.04, 1.54).

**Table 2:** Results of the meta-analysis on the association between occupational exposure to diesel exhaust and liver and pancreatic cancers

		Liver and biliary tract cancer				Pancreatic cancer			
Outcome	Stratum	n of studies	RR	95% CI	I^2^, p-value	n of studies	RR	95% CI	I^2^, p-value
Incidence and mortality	Sex								
	Male	13	1.07	0.96, 1.18	51.7%, 0.016	13	1.04	1.01, 1.08	0.0%, 0.598
	Female	2	0.97	0.75, 1.24	0.0%, 0.858	3	1.06	0.85, 1.33	0.0%, 0.744
	Region								
	North America	6	1.04	0.81, 1.35	56.4%, 0.043	7	1.13	0.94, 1.36	62.7%, 0.013
	Europe	8	1.07	0.97, 1.19	46.6%, 0.069	8	1.05	1.01, 1.09	0.0%, 0.701
	CASP score^†^								
	≤ median	8	1.16	0.90, 1.50	62.5%, 0.009	10	1.12	0.96, 1.30	49.1%, 0.039
	> median	6	1.04	0.98, 1.11	12.3%, 0.336	5	1.05	1.01, 1.09	0.0%, 0.569
	Publication year								
	Before 2000	8	1.27	1.04, 1.54	24.1%, 0.237	10	1.05	0.91, 1.20	20.1%, 0.258
	2000 or later	6	1.00	0.90, 1.10	54.5%, 0.051	5	1.09	1.00, 1.18	55.8%, 0.060
Incidence	Overall	7	1.09	0.99, 1.19	37.7%, 0.141	7	1.11	1.01, 1.22	55.2%, 0.037
	Sex								
	Male	6	1.10	0.995, 1.21	46.4%, 0.097	5	1.05	1.01, 1.09	0.0%, 0.539
	Female	2	0.97	0.75, 1.24	0.0%, 0.858	3	1.06	0.85, 1.33	0.0%, 0.744
	Region								
	North America	1	0.90	0.44, 1.86	na	2	1.46	1.20, 1.79	0.0%, 0.901
	Europe	6	1.10	0.99, 1.21	47.1%, 0.093	5	1.05	1.01, 1.09	0.0%, 0.503
	CASP score^†^								
	≤ median	2	1.55	1.14, 2.11	0.0%, 0.376	3	1.32	1.05, 1.66	29.3%, 0.243
	> median	5	1.05	1.003, 1.10	0.0%, 0.563	4	1.05	1.01, 1.09	0.0%, 0.418
	Publication year								
	Before 2000	4	1.41	1.09, 1.82	0.0%, 0.454	4	1.13	0.83, 1.55	22.2%, 0.278
	2000 or later	3	1.05	1.00, 1.11	10.7%, 0.326	3	1.10	1.00, 1.21	77.3%, 0.012
Mortality	Overall	9	1.02	0.84, 1.24	44.4%, 0.072	10	0.99	0.89, 1.09	0.0%, 0.589
	Sex								
	Male	9	1.02	0.84, 1.24	44.4%, 0.072	10	0.99	0.89, 1.09	0.0%, 0.589
	Female	0	nc			0	nc		
	Region								
	North America	5	1.06	0.80, 1.42	64.9%, 0.022	5	1.00	0.88, 1.13	11.3%, 0.341
	Europe	4	0.92	0.73, 1.14	0.0%, 0.470	5	1.00	0.79, 1.26	0.0%, 0.568
	CASP score^†^								
	≤ median	7	1.10	0.85, 1.42	52.7%, 0.048	8	1.00	0.89, 1.12	5.3%, 0.389
	> median	2	0.86	0.68, 1.09	0.0%, 0.706	2	0.98	0.75, 1.30	0.0%, 0.792
	Publication year								
	Before 2000	6	1.17	0.94, 1.45	13.0%, 0.332	8	0.97	0.87, 1.09	0.0%, 0.440
	2000 or later	2	0.84	0.67, 1.05	29.8%, 0.240	2	1.05	0.83, 1.33	0.0%, 0.637

RR: relative risk, CI: confidence interval, nc: not computable, na: not applicable, ^†^: median CASP score was equal to 10

Results were similar for liver cancer incidence (Table 2), with a suggestive positive association, albeit not significant (RR: 1.09; 95% CI: 0.99, 1.19). An association was also observed among male individuals (RR: 1.10; 95% CI: 0.99, 1.21). The association was not significant in the subgroup analyses according to the region where the included studies were conducted (Table 2), although the estimate was near-significant for those carried out in Europe (RR: 1.10; 95% CI: 0.99, 1.21). A positive significant association was instead found in subgroups according to CASP quality scores and for studies published before 2000 (Table 2).

Lastly, the analysis on mortality revealed no significant association, both overall and in the examined subgroups (Table 2).

Results showed a low or moderate degree of heterogeneity in most cases, including overall and subgroup analyses, and for all considered outcomes (Table 2).

**Fig. 3:**
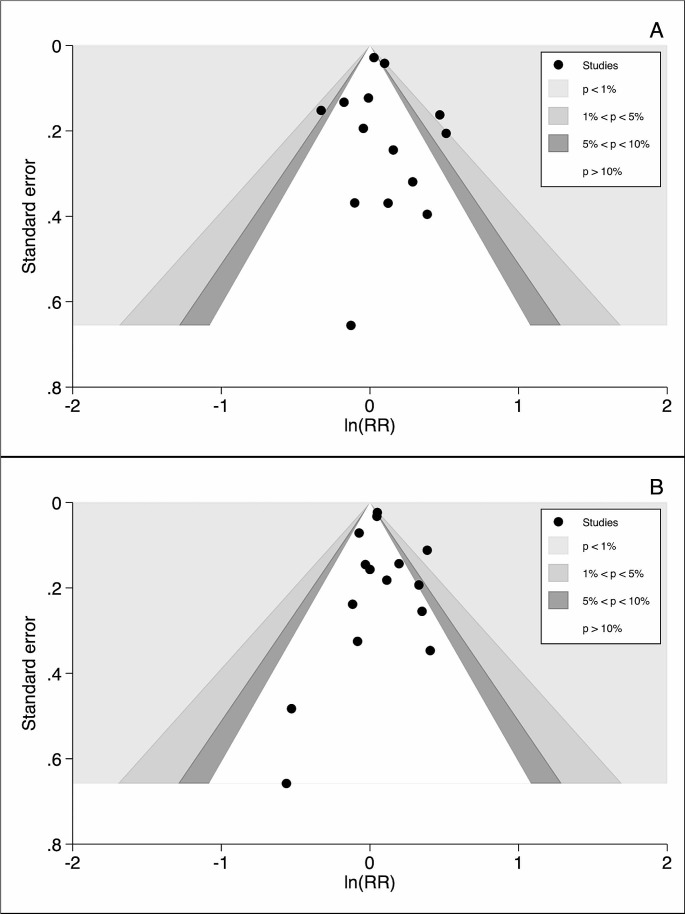
Contour-enhanced funnel plot to explore small-study effect for liver and biliary tract (box A) and pancreatic (box B) cancers, incidence and mortality combined

As for the assessment of publication bias, the contour-enhanced funnel plot (Fig. 3) showed slight asymmetry, although this was not paralleled by the result of Egger’s test (*p* = 0.66).

#### Pancreatic cancer

**Fig. 4:**
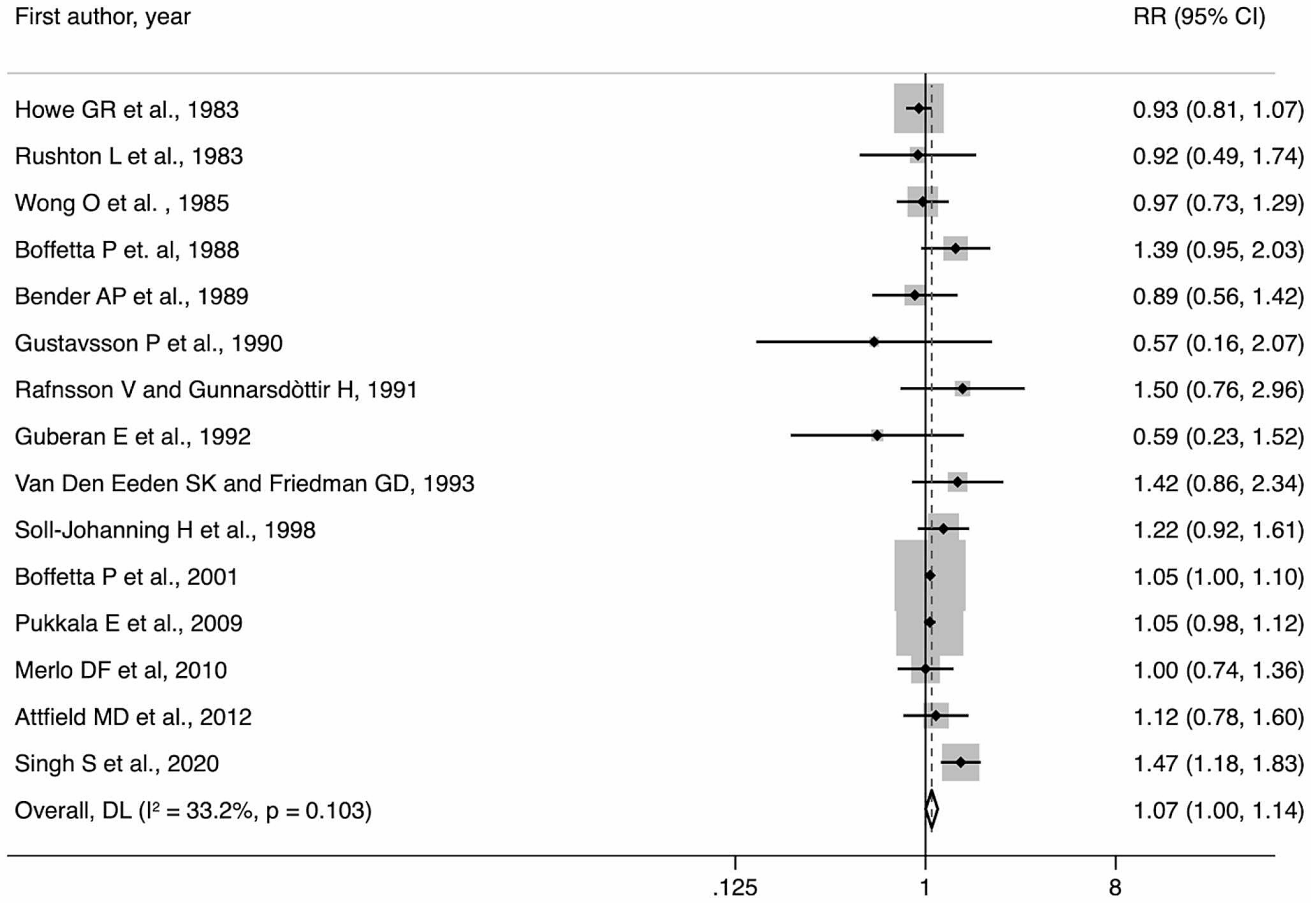
Results of the meta-analysis on the association between occupational exposure to diesel exhausts and pancreatic cancer incidence and mortality combined

The meta-analysis on incidence and mortality combined revealed a significant association between occupational DE exposure and risk of pancreatic cancer (Fig. 4, RR: 1.07, 95% CI: 1.00, 1.14). The leave-one-out analysis showed that results very not highly dependent upon a single study, although the confidence interval became wider when excluding some of them (Supplementary Fig. 2). In subgroup analyses (Table 2), a positive association was confirmed for male individuals (RR: 1.04, 95% CI: 1.01, 1.08), for studies conducted in Europe (RR: 1.05, 95% CI: 1.01, 1.09), and for those with a CASP score higher than the median value (RR: 1.05; 95% CI: 1.01, 1.09).

Findings on pancreatic cancer incidence were similar, both overall (RR: 1.11, 95% CI: 1.02, 1.22) and for male study participants (RR: 1.05, 95% CI: 1.01, 1.09). However, when stratifying according to the study region, studies carried out in America had a higher pooled risk estimate than those conducted in Europe (Table 2), albeit only two studies were in the former group. The association remained significant in CASP score subgroups.

Instead, as for pancreatic cancer mortality, meta-analytic estimates did not show an association with occupational DE exposure (Table 2).

Low levels of heterogeneity were observed overall and in most of the subgroup analyses (Table 2).

Lastly, a certain degree of asymmetry was evident from visual inspection of the funnel plot (Fig. 3), but the result of Egger’s test suggested no occurrence of small-study effect (*p* = 0.66).

## Discussion

The findings of our study suggest that occupational exposure to DE is associated with pancreatic cancer. This association was mainly driven by an increase in incidence-based studies, especially among male individuals, while no marked variations in estimates according to study region or CASP score were observed. As for liver cancer, even though pooled estimates were suggestive of a positive association for incidence, they did not reach significance, with exceptions being represented by the results from the subgroup analysis according to CASP scores and by the estimate based on studies published before 2000. Perhaps, the latter might be explained by technical and industrial progress that is progressively leading to a reduction of emissions from diesel engines over time, hence reducing liver cancer risk among exposed workers. However, the opposite was true for pancreatic cancer, thus other factors might actually explain observed differences according to publication year, including perhaps differences in study design and methodology. In addition, chance might as well be responsible of these conflicting results.

After ingestion or inhalation, small-sized DE particles might enter the bloodstream and be deposited in the liver [50, 51], as well as reach the pancreas [52]. Hence, DE particles might increase the risk of liver and pancreatic cancers through a number of mechanisms, including DNA damage, oxidative stress, and inflammation [52–54]. In addition, among chemicals contained in DE are polycyclic aromatic hydrocarbons [12], which have been reported to be stored in pancreatic tissue in humans [55], and occupational exposure to them has been suggested to be associated with increased risk of pancreatic cancer, although with non-conclusive evidence [9]. 

Two previous meta-analyses, published in 2000 and 2014, investigated the association between occupational DE exposure and pancreatic cancer, with results in contrast with our findings. Both of them, indeed, observed no significant association. The first one by Ojajärvi et al. did not report results by study design and found an overall pooled RR estimate of 1.0 (95% CI: 0.9, 1.3; n of studies: 5) among men [19], while the second one reported a RR of 1.03 (95% CI: 0.93, 1.13; n of studies: 9) for cohort studies only [20]. To the best of our knowledge, instead, no meta-analyses evaluating the relationship between occupational DE exposure and liver cancer have been conducted so far.

Among the main limitations of our meta-analysis there is the lack of consideration of potential confounders in the primary studies. For instance, just a few of them controlled for tobacco smoking either through adjustment or standardization [34, 45] and none for alcohol drinking. Both tobacco smoking and alcohol drinking are risk factors for liver and pancreatic cancers [56–58], hence potentially being responsible for the observed significant results. In this regard, the limited number of studies that included such information in the analysis prevented us from carrying out related subgroup or sensitivity analyses. Additionally, included studies did not directly evaluate DE exposure at the individual level and did not carry out environmental measurements, but DE exposure was rather assessed based on the working categories. Also, levels of exposure could be supposed to vary between cohorts, according to different participants’ occupation and preventive measures that could have been adopted in the workplace, including personal protective equipment [18, 59]. In this regard, it should be noted that, even within the same cohort of individuals with the same occupation, some variability in terms of exposure could be expected, for instance according to differences in tasks carried out by the participants. Furthermore, lack of data did not allow us to take into account several additional aspects related to exposure, such as duration of exposure and employment, time since cessation of exposure, and its intensity, hence it was not possible to evaluate the occurrence of a dose-response relationship between the exposure and the considered outcomes. Additionally, it was not possible to rule out potential co-exposure with other occupational carcinogens, since studies included in this review did not report related information. Also, it should be noted that certain categories of workers exposed to diesel might also experience exposure to other environmental pollutants not necessarily related to DE emissions, such as those deriving from road traffic for professional drivers, which could thus confound results of our meta-analysis. Moreover, although occupational exposure to diesel might have higher intensity and duration for specific occupations, the general population has been exposed to DE too over recent decades given the widespread use of vehicles adopting this type of fuel, especially in Europe [60]. Thus, external populations used as a reference in most of the studies included in our meta-analysis may have also had substantial exposure to DE emissions, albeit not in occupational settings and likely with lower cumulative duration and intensity. Furthermore, since only a limited number of studies reported separate results on liver and gallbladder cancers, we did not analyze them separately. Additionally, our search strategy was based on the use of a single electronic database to retrieve papers eligible for inclusion in our meta-analysis, perhaps leading us to miss additional relevant studies. Moreover, all the studies included in our review were carried out in Europe or North America, thus suggesting the need for further studies from other regions, such as Africa and Asia, where liver cancer incidence is especially high [5]. Due to lack of data, it was also not possible to evaluate whether DE exposure has a different strength of association with either liver or pancreatic cancer according to study participants’ ethnicity.

In summary, our results suggest that, although weakly, occupational exposure to diesel might be associated with pancreatic cancer risk, while findings on liver cancer were only suggestive of a similar relationship, but inconclusive. However, these results should be taken cautiously, given the abovementioned important limitations of the studies included in the meta-analysis. Hence, further high-quality prospective studies appropriately considering potential confounders, with periodic or continuous environmental measurements, and evaluating actual occupational DE exposure at the individual level are warranted to clarify its effect as regards to liver and pancreatic cancers risk.

### Electronic supplementary material

Below is the link to the electronic supplementary material.

Supplementary Material 1

## Data Availability

All data used for this study are available from the first Author.

## Code availability

The software code used for this study is available from the first Author.
