# Supplementary material for: Occupational exposure to diesel exhausts and liver and pancreatic cancers: a systematic review and meta-analysis
Source: Eur J Epidemiol. 2024 Jan 30;39(3):241–55. doi: 10.1007/s10654-024-01099-4 (PMC10995068; doi:10.1007/s10654-024-01099-4)

SUPPLEMENTARY MATERIAL

Occupational exposure to diesel exhausts and liver and pancreatic cancers: a systematic review and meta-analysis

Michele Sassano[1], Giulia Collatuzzo[1], Federica Teglia[1], Paolo Boffetta[1,2,3]

[1] Department of Medical and Surgical Sciences, University of Bologna, Bologna, Italy.

[2] Stony Brook Cancer Center, Stony Brook University, Stony Brook, New York, USA.

[3] Department of Family, Population and Preventive Medicine, Renaissance School of Medicine, Stony Brook University, Stony Brook, NY, USA

Correspondence:
Paolo Boffetta
Department of Medical and Surgical Sciences, University of Bologna, Bologna, Italy.
paolo.boffetta@unibo.it

Supplementary Table 1. Modified version of the Critical Appraisal Skills Programme (CASP) checklist for cohort studies used for quality assessment.

Supplementary Figure 1. Leave-one-out meta-analysis for the association between occupational exposure to diesel exhausts and liver and biliary tract cancer incidence and mortality combined.

Supplementary Figure 2. Leave-one-out meta-analysis for the association between occupational exposure to diesel exhausts and pancreatic cancer incidence and mortality combined.

Supplementary Table 1. Modified version of the Critical Appraisal Skills Programme (CASP) checklist for cohort studies used for quality assessment.

Items	Possible scores
**Section A: Are the results of the study valid?**	
1. Did the study address a clearly focused issue?	- 1.5<br>- 1.0<br>- 0.0
2. Was the cohort recruited in an acceptable way?	- 1.5<br>- 1.0<br>- 0.0
3. Was the exposure accurately measured to minimise bias?	- 1.0<br>- 0.5<br>- 0.0
4. Was the outcome accurately measured to minimise bias?	- 1.0<br>- 0.5<br>- 0.0
5.(a) Have the authors identified all important confounding factors?	- 1.0<br>- 0.5<br>- 0.0
5.(b) Have they taken account of the confounding factors in the design and/or analysis?	- 1.0<br>- 0.5<br>- 0.0
6.(a) Was the follow up of subjects complete enough?	- 1.0<br>- 0.5<br>- 0.0
6. (b) Was the follow up of subjects long enough?	- 1.0<br>- 0.5<br>- 0.0
**Section B: What are the results?**	
7. What are the results of this study?	Excluded
8. How precise are the results?	- 1.0<br>- 0.5<br>- 0.0
9. Do you believe the results?	- 1.0<br>- 0.5<br>- 0.0
**Section C: Will the results help locally?**	
10. Can the results be applied to the local population?	- 1.0<br>- 0.5<br>- 0.0
11. Do the results of this study fit with other available evidence?	- 1.0<br>- 0.5<br>- 0.0
12. What are the implications of this study for practice?	- 1.0<br>- 0.5<br>- 0.0

For each item, scores were assigned according to researchers' consideration of the quality of the content (higher score means higher quality).

Supplementary Figure 1. Leave-one-out meta-analysis for the association between occupational exposure to diesel exhausts and liver and biliary tract cancer incidence and mortality combined.

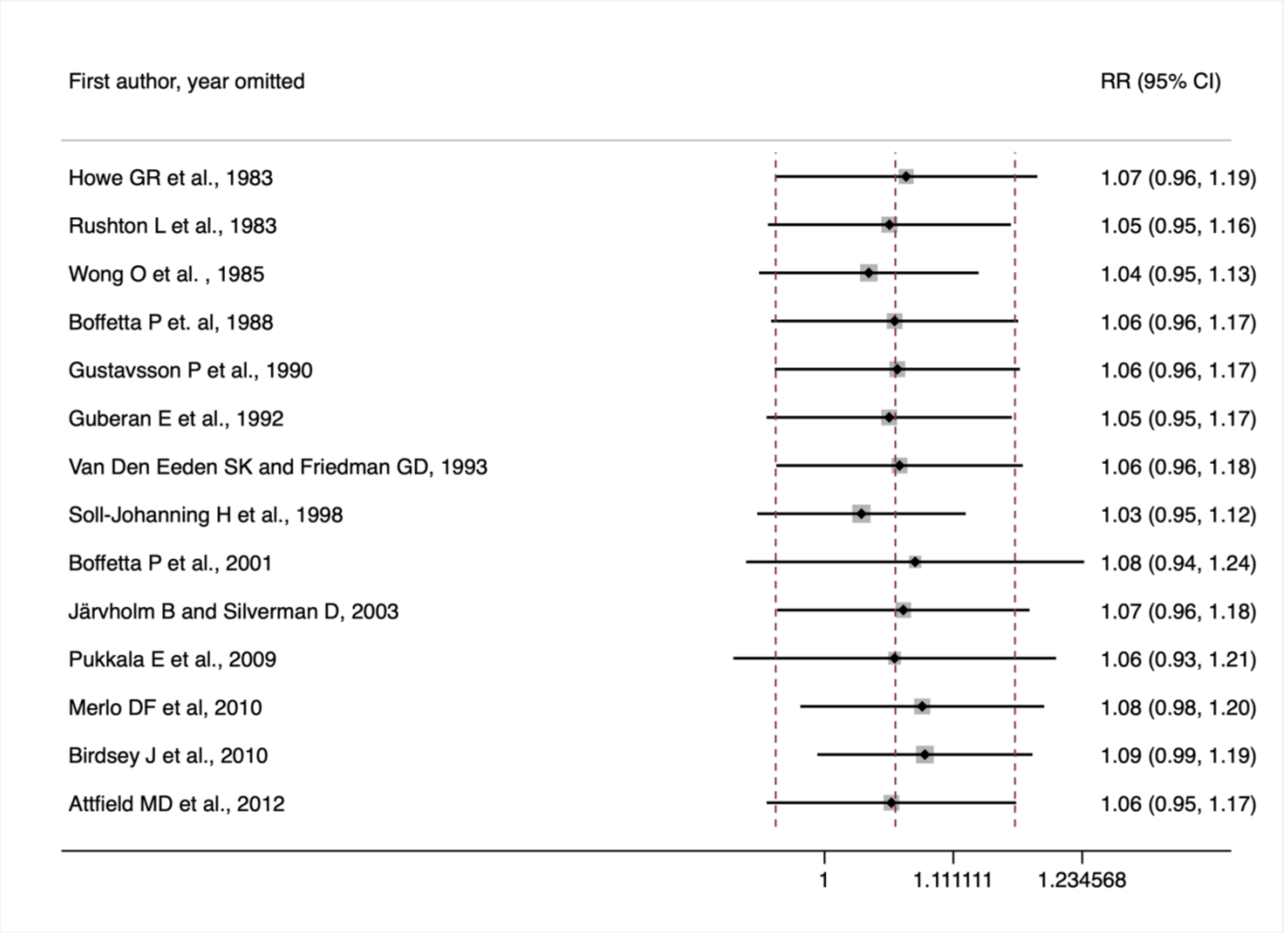

Supplementary Figure 2. Leave-one-out meta-analysis for the association between occupational exposure to diesel exhausts and pancreatic cancer incidence and mortality combined.

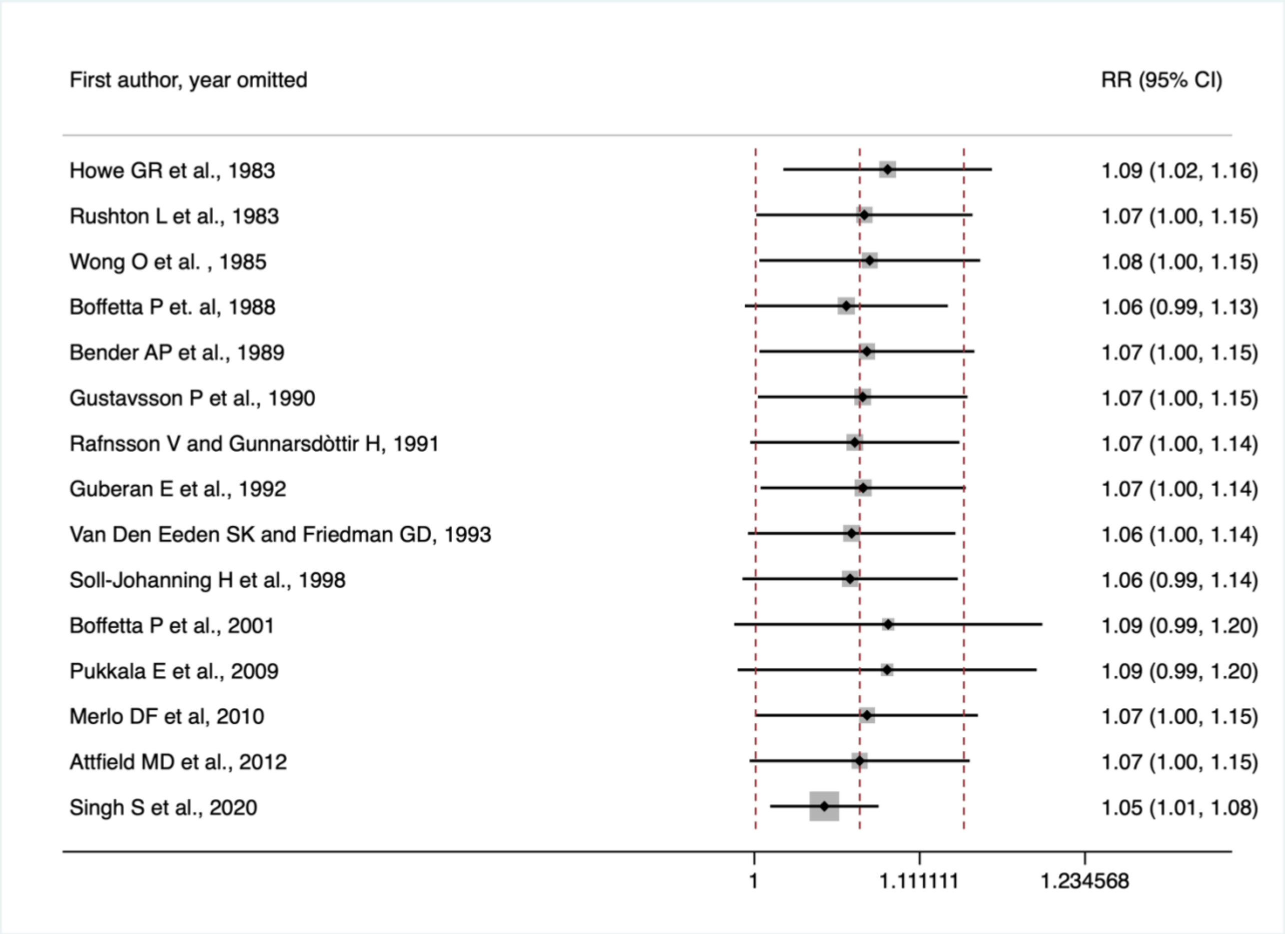

Supplement: Supplementary file 1 — Supplementary Material 1 [file 10654_2024_1099_MOESM1_ESM.pdf]
